# Potential drug mechanism(s) targeting the contractile status of hepatic stellate cells

**DOI:** 10.3389/fphar.2012.00187

**Published:** 2012-10-25

**Authors:** Claudio Bucolo, Filippo Drago, Salvatore Salomone

**Affiliations:** Department of Clinical and Molecular Biomedicine, Section of Pharmacology and Biochemistry, University of CataniaCatania, Italy

Hepatic stellate cells (HSCs) represent the major cell type involved in liver fibrosis. In normal liver HSCs are in a quiescent state; when the liver is damaged, stellate cells can change into an activated state, characterized by proliferation, contractility, chemotaxis, and secretion of extracellular matrix proteins, such as collagen, which can lead to cirrhosis (Li et al., 2008). HSCs are considered as “hepatic pericytes,” because they possess protrusions that wrap around the sinusoids and their contraction contribute to the modulation of sinusoidal resistance and blood flow (Soon and Yee, 2008). Several agonists, either paracrine or circulating, stimulate HSC contraction; these include angiotensin-II (ATII), endothelin-1 (ET-1), arginine-vasopressin, thrombin, eicosanoids, and catecholamines. Circulating levels of these agents may be elevated in patients with liver disease, and increased in animal models of liver injury. In particular, perfusion of isolated rodent livers with ATII or ET-1 causes a reduction in sinusoidal diameter associated to increase in portal pressure, while administration of ATII or ET-1 receptor antagonists decreases portal pressure (Farrell et al., 2008; Reynaert et al., 2008). This evidence underscores the role of agonists that increase HSC contractility in the regulation of hepatic blood flow. On the other hand, several agents, including nitric oxide, carbon monoxide, and prostaglandins, may counteract the effects of contraction-inducing stimuli by causing HSC relaxation. Nitric oxide production is reduced in the injured liver, while nitric oxide donors reduce portal pressure induced by contractile stimuli in perfused liver (Farrell et al., 2003; Laleman et al., 2007). Thus, current view considers sinusoidal tone as finely modulated by the balance between HSC relaxation and HSC contraction. Regulation of contractility status in HSC recapitulates the general mechanism well known in vascular smooth muscle cells (VSMC). In HSC, myosin light chain phosphorylation activates myosin II and supports contraction, whereas reduction of myosin phosphorylation inhibits contractile force generation. Cytosolic Ca^2+^ signaling may regulate HSC contraction by activating myosin light chain kinase, which selectively phosphorylates the myosin regulatory light chain. Available data, however, indicate that the contribution of Ca^2+^ signaling to the regulation of HSC contraction might be less important than in VSMC. Instead, a critical signaling pathway regulating myosin phosphorylation in HSC seems to be RhoA/Rho kinase. Rho-kinase (ROK) is a cytosolic kinase activated by the small GTPase RhoA, linking different vasoactive receptors to the myosin light chain phosphatase (MLCP). Activation of ROK inhibits the activity of MLCP and thereby increases phosphorylation of myosin light chains. In liver cirrhosis intrahepatic ROK is upregulated and inhibition of ROK decreases hepatic-portal resistance and portal pressure (Hendrickson et al., 2012).

Nonalcoholic fatty liver disease (NAFLD) is a relatively common condition, characterized by fatty accumulation (steatosis) in the liver and related to insulin resistance and metabolic syndrome, that often progresses into the more serious non-alcoholic steato-hepatitis (NASH) and, in some cases, to cirrhosis or hepatocarcinoma. The transition from NAFLD to NASH depends on a superimposed inflammatory mechanism, that induces activation of HSC, injury to hepatic microcirculation, venous obstruction, increased production of extracellular matrix, and fibrous septation, (Wanless and Shiota, 2004; Bian and Ma, 2012). Activation of HSC and subsequent vascular insult is recognized as an important pathogenic step. Both non-pharmacological and pharmacological treatments have been proposed for NAFLD and NASH, but no drug therapies have been so far accepted as standard therapy. Non-pharmacological treatment includes measures to gradually reduce body weight such as diet, aerobic exercise, and bariatric surgery. Drug treatment includes chiefly insulin sensitizers such as metformin and thiazolidinediones (Musso et al., 2012). Other drugs, that are not primarily acting on liver metabolic activity, such as angiotensin receptor blockers (ARBs), have been also proposed (Yokohama et al., 2004). The theoretical mechanisms underlying the effectiveness of such drug therapies are obviously diverse. But what we want to point here is the potential relevance of HSCs as pharmacological target, particularly regarding their role in regulating the caliber of hepatic sinusoids and thereby portal blood flow, perfusion pressure, and resistance. Activation of peroxisome proliferator-activated receptor gamma (PPARγ) inhibits HSC collagen production and modulates HSC adipogenic phenotype at transcriptional and epigenetic levels (Zhang et al., 2012). The ability of activating PPARγ-dependent gene expression is shared by thiazolidinediones and at least some ARBs, such as Telmisartan and Irbesartan (Schupp et al., 2004). It seems therefore plausible that these two classes of drugs may share a PPARγ-dependent action on HSC, resulting in a non-fibrogenic quiescent phenotype. Moreover, besides PPARγ-mediated effects, thiazolidinediones have been reported to exert PPARγ-independent effects on smooth muscle cells and vascular tone (Salomone, 2011; Salomone and Drago, 2012) that might be exerted also on HSC. In particular, PPARγ ligands inhibit Rho/ROK pathway in vascular tissues, by inducing the expression of protein tyrosine phosphatase SHP-2 (Wakino et al., 2004) and cause a rapid inhibition of myosin phosphatase target subunit 1 (MYPT1) phosphorylation in a ROK-independent manner (Atkins et al., 2009). Inhibitors of the renin-angiotensin system, including ARBs, counteract liver fibrosis, and reduce portal hypertension. The main effect of ARBs is as antagonists of the AT_1_ receptor, thereby inhibiting transformation of the quiescent HSC into the myofibroblast like activated HSC and the synthesis of transforming growth factor-beta1, the major profibrotic cytokine in the liver (Tox and Steffen, 2006). ARBs obviously also oppose the effect of ATII on HSC contractility. Thiazolidinediones and ARBs therefore both inhibit the transition of HSC to fibroblast-like phenotype, responsible for matrix deposition and cirrhotic outcome and decrease the contractility status of HSC, which may have an additional effect on portal pressure and portal blood flow. Telmisartan, beside the ATII-dependent vascular effect, induces also, at least *in vitro*, an endothelium-dependent vasodilatation (Siarkos et al., 2011), that may further impact hepatic blood flow in NAFLD/NASH. Interestingly, a recent study using high-content analysis in a rat model predicts telmisartan and pioglitazone as very effective antifibrotic drugs (Zheng et al., 2011).

In conclusion, we outline here the importance of two drug mechanisms targeting HSC that may be relevant when treating NAFLD/NASH: PPARγ-stimulation and relaxation of contractile apparatus. These two mechanisms are likely to have different kinetics, the first requiring gene-expression, the second occurring as soon as the drug binds membrane receptors and/or transduction proteins that affect myosin phosphorylation. Therefore, beside the opposing effect on HSC activation (such that dependent from PPARγ), we propose that studies should be designed to examine *in vitro* the effects of isolated drugs on HSC contractility and/or, *in vivo*, their effect on portal blood pressure and flow, as predictors of efficacy for NAFLD/NASH treatment in preclinical settings.
